# Posterior Interosseous Nerve Syndrome Resulting from Parosteal Lipoma of the Proximal Radius: An Elusive Diagnosis Yet Excellent Outcome

**Published:** 2017-01

**Authors:** Muhammad Saaiq, Saad Siddiui

**Affiliations:** Department of Plastic Surgery, Pakistan Institute of Medical Sciences (PIMS), Shaheed Zulfiqar Ali Bhutto Medical University (SZABMU), Islamabad-44000, Pakistan

**Keywords:** Posterior interosseous nerve, Posterior interosseous nerve syndrome, Parosteal lipoma

## Abstract

A 53-year old man presented with seven months history of progressive weakness of extension of the digits and the thumb of the left hand. The wrist extension was normal and sensations were also intact. The patient had also been noticing a progressively enlarging lump on the lower anterolateral aspect of the left antecubital fossa for the last three months. Physical examination andelectro diagnostic studies revealed motor deficit along the posterior interosseous nerve (PIN) distribution with preservation of sensations. Also a soft tissue solitary lump (measuring 6×5 cm in its greatest dimensions) was palpable in the left antecubital fossa. The magnetic resonance imaging (MRI) of the forearm revealed a well-defined, non-enhancing, homogenous, fat intensity lesion in the left antecubital fossa, attached to the proximal radius. The patient underwent surgical excision of the lump with decompression of the PIN in the radial tunnel. Histopathology confirmed the diagnosis of parosteal lipoma. Although the diagnosis was elusive at the very outset, yet prudent clinical judgment, appropriate ancillary investigations and timely surgical intervention resulted in optimal functional recovery of the hand drop. There was complete motor recovery at 4-months follow up with no recurrence of the lipomaafter one year.

## INTRODUCTION

Parosteal lipoma is an extremely rare benign neoplasm composed of mature adipose tissue contiguous with underlying periosteal bone. It accounts for 0.3% of all lipomas.^1^^-^^3^ It affects the proximal radius, humerus, femur, tibia, clavicle, ribs and the pelvis. The inciting cause is often unknown. The age group frequently affected is 40-60 years. The vast majority of patients present with a history of a slow-growing, painless and non-tender lump. Motor and sensory disturbances from compression of the adjacent nerves may occur during the course of its progression.^3^^-^^6^

The posterior interosseous nerve (PIN) arises from the bifurcation of the radial nerve in the proximal forearm. It is a pure motor nerve that innervates extensors of the forearm (with the exception of the extensor carpi radialis longus (ECRL), brachioradialis, or anconeus) and the abductor pollicis longus (APL). Immediately distal to the bifurcation, the PIN travels through the radial tunnel, which is a 5-cm space dorsally floored by the capsule of the radiocapitellar joint, laterally bounded by the ECRL and extensor carpi radialis brevis (ECRB) muscles, medially bordered by the biceps tendon and brachialis muscle and volarly covered by the brachioradialis.^6^^-^^9^

Within the confined space of the radial tunnel, there are five potential sites of PIN compression; i.e. a- the fibrous bands to the radiocapitellar joint between the brachialis and brachioradialis; b- the recurrent radial vessels (i.e. leash of Henry); c- the proximal edge of the ECRB; d- the proximal edge of the supinator (i.e. arcade of Fröhse); and e- the distal edge of the supinator. Compression of the PIN gives rise to either of the two distinct compression syndromes. i.e. The posterior interosseous nerve syndrome (PINS) and the radial tunnel syndromes (RTS). In contrast to PINS, the RTS is characterized by lateral proximal forearm pain with no discernible motor weakness. The management for both is the same.^6^^-^^9^ To the best of our knowledge, there are only a handful of cases of PINS secondary to parosteal lipoma of the proximal radius reported in the published literature. Ours is the first case to be reported in this regard from any plastic surgical facility from Pakistan. This rarity prompts us to share our management experience.

## CASE REPORT

A 53-year old Pakistani male patient presented to our unit with seven months history of weakness of extension of the fingers and the thumb of the left hand. The difficulty in extension was of gradual onset and progressively worsening with the resultant gradual loss of manual dexterity. The wrist extension was normal, albeit with mild radial deviation of the hand on attempted extension. The patient was particularly concerned about his progressive hand drop. He also had been noticing a progressively enlarging lump on the lower anterolateral aspect of the left antecubital fossa for the last three months. The lump was painless, soft and slow growing. The patient had no history of any traumatic insult to the affected limb or any other concomitant illness. 

Neurological examination of the affected limb was performed. On the Medical Research Council scale (MRC scale), the power scores for the triceps, brachioradialis, and ECRL were all 5/5, however the scores for the ECRB, extensor digitorum communis, extensor carpi ulnaris, extensor pollicis longus and brevis, extensor indicis proprius, extensor digiti minimi and APL were all 2/5. The forearm supination was also weakened. There was no sensory deficit. There was a solitary, soft, well-defined lump in the left antecubital fossa, measuring approximately 6×5 cm in size. The lump was relatively fixed while the overlying skin was mobile. Positive Tinel signal on the PIN was elicit-able on tapping over the lump.

**Fig. 1:**
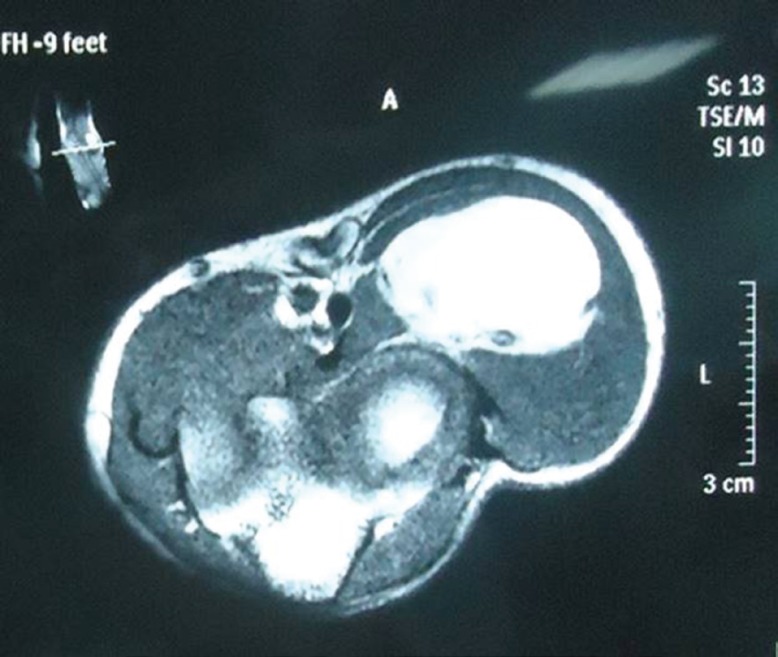
MRI (transverse section) showing relationship of the parosteal lipoma to the underlying radius and the adjacent muscles of the forearm

**Fig. 2:**
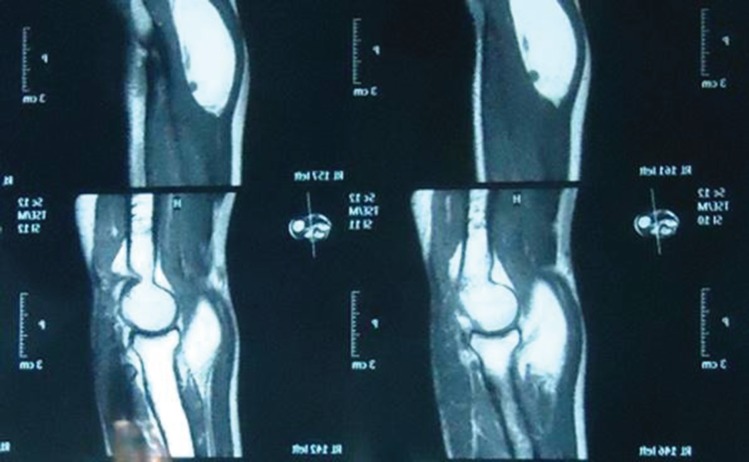
MRI (coronal section) showing relationship of the parosteal lipoma to the underlying radius and the adjacent muscles of the forearm

**Fig. 3:**
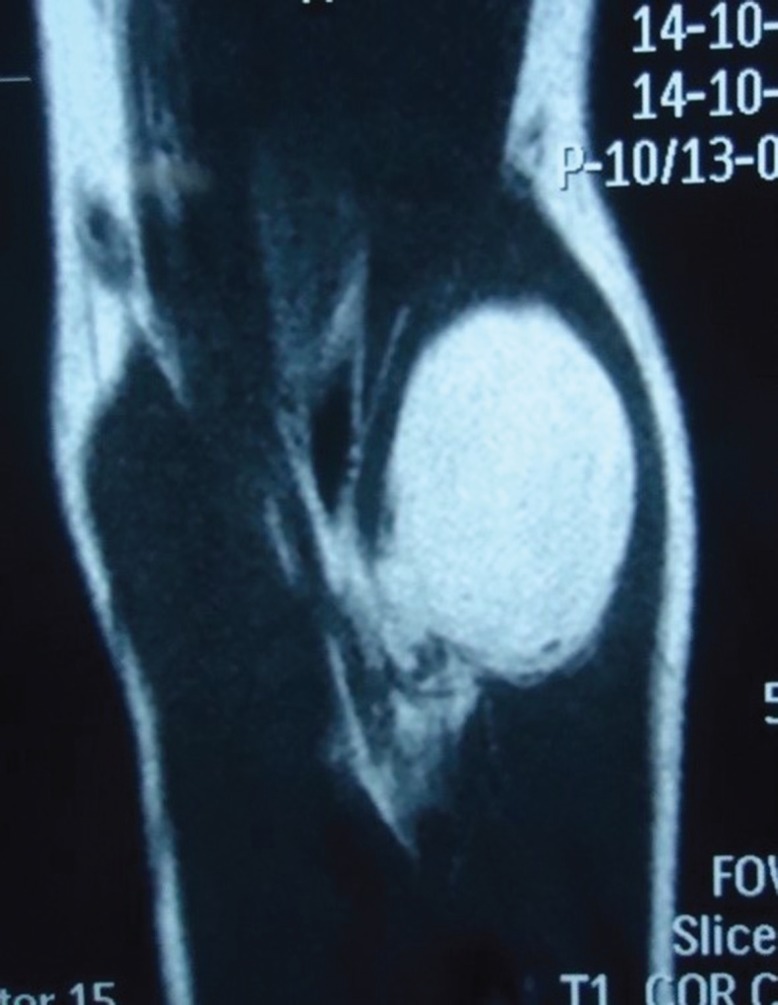
MRI (coronal section) showing relationship of the parosteal lipoma to the underlying radius and the adjacent muscles of the forearm

Electromyography and nerve conduction studies revealed motor deficit of the PIN, consistent with the aforementioned findings on physical examination. The MRI of the forearm revealed a well-defined, non-enhancing, homogenous, fat intensity lesion in the left antecubital fossa. It was completely separate from the adjacent forearm muscles and attached to the proximal radius (Figures 1-3). The patient had already received several months of conservative management including the hand rest, avoidance of aggravating activities, nocturnal splinting, stretching/ exercises, and a variety of non-steroidal anti-inflammatory drugs but had no improvement.

We undertook surgical exploration and excision of the lump under general anesthesia. An anterior approach was employed involving a curvilinear incision beginning proximal to the lateral epicondyle and continuing in the interval between the biceps and brachioradialis muscles and then curving 2 cm above the elbow flexion crease over the mobile wad and then medial to the border of the brachioradialis. The fascia along the brachioradialis muscle was divided and the muscle retracted laterally while retracting the biceps and pronator teres medially. The lump was carefully mobilized and excised by undocking the periosteal attachment. 

**Fig. 4:**
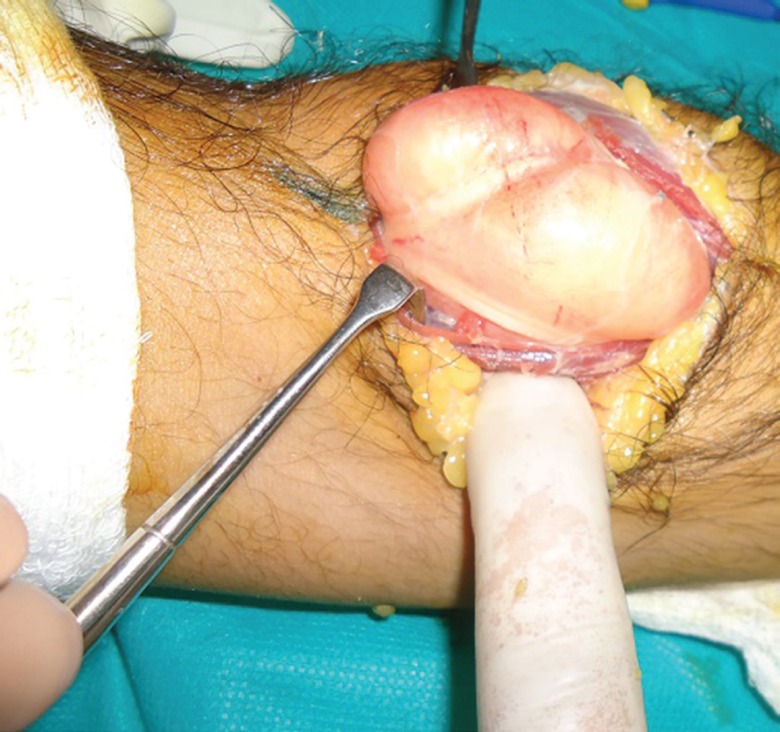
Intraoperative photograph showing the parosteal lipoma. The overlying stretched medial and lateral branches of the PIN are clearly visible

**Fig. 5:**
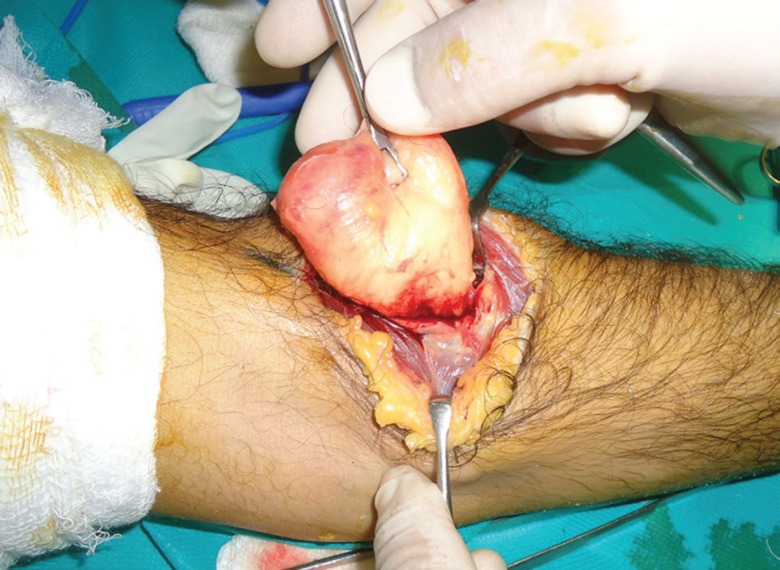
Intraoperative photograph showing the parosteal lipoma. The PIN branches have been carefully separated, retracted and lipoma mobilized with proximal radial attachment still in situ

**Fig. 6:**
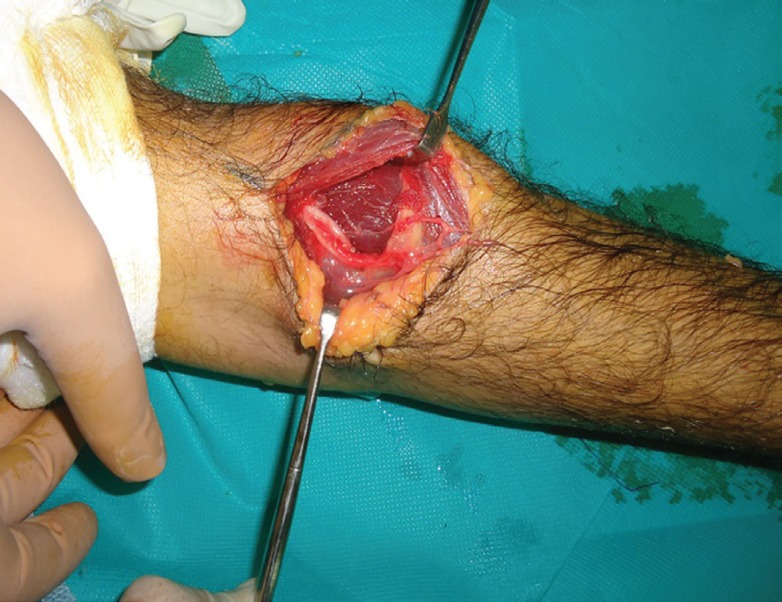
Intraoperative photograph after the parosteal lipoma has been excised and all potential sites of PIN compression have been released. The overlying stretched branches of the PIN are now lying un-stretched

Care was taken to safeguard the overlying stretched branches of the PIN. Only bipolar electric cautery was used during the dissection to avoid any further nerve damage. Once the lump was removed, the nerve branches were traced distally in the interval between the brachialis and brachioradialis and their integrity confirmed visually. The fibrous bands overlying the PIN and the edge of the ECRB were released. The arcade of Fröhse was divided. The mobile wad muscles were retracted to expose the superficial head of the supinator, which was then divided. Thus the adequacy of PIN decompression was ensured by releasing all the potential sites of PIN compression. After ensuring adequate hemostasis the wound was closed in layers (Figures 4-6). 

**Fig. 7:**
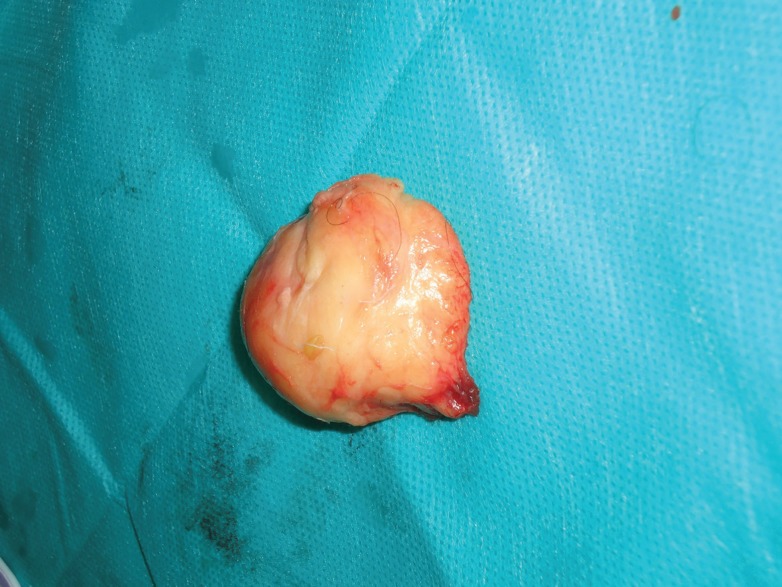
Photograph showing the excised parosteal lipoma specimen (measuring approximately 6×5×4 cm size) the peripheral fat component and the bony attachment site are visible

On gross pathology examination, the excised lump was a well circumscribed mass measuring 6×5×4 cm in size (Figure 7). The cut surface was soft, yellowish and homogenously greasy. On histopathology examination, both the peripheral fat as well as the bony attachment were examined microscopically and found be composed of mature lipocytes and the features were consistent with the diagnosis of parosteal lipoma. There was no cellular atypia or lipoblasts. 

## DISCUSSION

In our patient the PINS was caused by a parosteal lipoma of the proximal radius. The published literature has highlighted three major groups of causes of non-traumatic isolated PIN palsy. These include space-occupying lesions (SOLs), the supinator syndrome, and isolated neuritis. It is important to differentiate between these causes because the treatment differs accordingly. A variety of SOLs have been reported in the literature and include lipomas, ganglia, intracapsular chondromas, synovial hemangiomas, myxomas, and synovial chondromatosis. The supinator syndrome occurs when the PIN is compressed by the proximal boundary of the supinator muscle.^10^^-^^12^

In our patient we found drop of all the digits and the thumb of the affected left hand as both the medial and lateral branches of the PIN were unduly stretched by the underlying parosteal lipoma. We termed it as “hand drop” in order to differentiate it from wrist drop of the radial nerve palsy. Suematsu Netal^6^ have classified PIN palsy into three types of (i) Type 1 (drop of fingers and thumb) caused by a simultaneous compression of the recurrent branch and the descending branch of PIN at the entrance point and within the supinator; (ii) Type 2 (drop of fingers) caused by compression of the recurrent branch only; and (iii) Type 3 (drop thumb) caused by compression of the descending branch only.^6^

In our patient we performed MRI scan as it is superior to the CT scan for the evaluating a parosteal lipoma. The lesion is identified on the MRI as a juxta-cortical mass with signal intensity identical to that of subcutaneous fat, regardless of the pulse sequence. Heterogeneity in these lesions may be present and corresponds to the pathologic components in the lesion. Areas with intermediate signal intensity on T1-weighted images that are high signal intensity on T2-weighted images will represent the cartilaginous components in parosteal lipoma. Fibrovascular septae may cause a lobulated appearance of the fat component, with low-signal-intensity strands on T1-weighted images that become higher in signal intensity on the long TR images (particularly with fat suppression).^13^^-^^15^

Larger areas of bone production surrounded by the lipomatous components will also well demonstrated on MRI. Adjacent muscle atrophy, poorly demonstrated by CT scan, is better identified on MRI as increased striations of fat in the affected muscle and may be caused in part by the associated nerve entrapment. This finding is best appreciated on T2-weighted images because of the decreased signal intensity of normal muscle relative to fat. The MRI also best demonstrates the relationship of the lesion to the underlying native bone and the adjacent muscles and this information is important for surgical planning as the parosteal lipoma is usually firmly adherent to the underlying cortex at the site of surface bone production.^13^^-^^15^

The definitive treatment of parosteal lipoma causing PINS is complete surgical excision of the lipoma, as we did in our patient. We carefully separated and retracted the overlying stretched branches of the PIN to ensure compete excision without causing any further damage to the nerve. Also we carefully employed bipolar cautery to avoid any risk to the nerve by monopolar cautery. The bony attachment of the parosteal lipoma to the proximal radius was separated using subperiosteal dissection with the help of an osteotome. The published literature has described surgical removal of the bony stalk of a parosteal lipoma by using either a subperiosteal dissection (which entails the separation of the lesion from the underlying bone using an osteotome) or segmental resection of the source bone. This latter part of the surgical excision is an additional step to the relatively easy dissection usually performed for a soft tissue lipoma elsewhere in the body.^1^^,^^5^

We observed complete motor recovery of the PIN four months post operatively. Our outcome results conform to Hashizume *et* *al.*^16^ who have also reported full recovery in 24 out of 25 patients who underwent surgical decompression of the PIN. However Vrieling *et* *al.*^17^ have reported relatively less favorable results among their patients. The difference in the reported outcomes is probably due to variability in the timing of the surgical intervention. Intuitively early surgical intervention is crucial for achieving optimal outcome. If surgery is unduly delayed for approximately 18 months, fibrosis of the affected muscles may occur, making tendon transfers the only viable option for achieving functional restoration.^17^

Although the diagnosis of parosteal lipoma causing PINS happens to be an elusive one at the very outset, yet prudent clinical judgment, use of appropriate ancillary investigations and timely surgical intervention results in optimal functional recovery of the hand drop. Any patient presenting with hand drop (i.e. inability to extend the digits and thumb) with intact wrist extension and intact sensations, should be suspected of having PINS and a careful search for possible etiology in the cubital fossa be performed.

## CONFLICT OF INTEREST

The authors declare no conflict of interest.
